# Exploring the genetic link between type 2 diabetes and its complications and esophageal malignancy: A Mendelian randomization study

**DOI:** 10.1097/MD.0000000000047202

**Published:** 2026-01-16

**Authors:** Mingzhi Lin, Chongrui Li, Bin Li, Zhongting Li, Yiming Hui, Haitian Li, Yuzhen Chen, Zhizhong Zheng

**Affiliations:** a Department of Thoracic Surgery, The Second Hospital and Clinical Medical School, Lanzhou University, Lanzhou, Gansu Province, China; b School of Clinical Medicine, Jining Medical University, Jining, China.

**Keywords:** causality, esophageal malignancy, multivariate Mendelian randomization, Mendelian randomization, type 2 diabetes

## Abstract

Type 2 diabetes (T2D) is an important risk factor for a range of GI malignancies. Nevertheless, the causal relationship between T2D and esophageal malignancies remains to be elucidated. Furthermore, the mechanisms leading to progression from T2D to esophageal malignancy have not been clearly characterized. The purpose of this study was to conduct a Mendelian randomization (MR) study to investigate the causal effect of T2D and its complications on the development of esophageal malignancies. In addition, this study aimed to perform a multivariate MR analysis to exclude potential confounders in this association. Genetic variation was used as an instrumental variable for T2D. Pooled data on T2D, as well as on T2D with complications and esophageal malignancies, were obtained from the European Bioinformatics Institute and Finnish databases. The study was conducted using 2-sample MR, multivariate MR. The study revealed a causal relationship between T2D (N = 4,90,089), T2D with comorbidities and esophageal malignancies (N = 1,74,238; T2D: odds ratio = 1.36, 95% confidence interval: 1.07–1.72, *P* = .01). Even after adjusting for confounders such as body mass index and hypertension, T2D remained statistically significant (odds ratio = 1.31, 95% confidence interval: 1.02–1.69, *P* = .036). The present MR study supports T2D as a causal risk factor for esophageal malignancy. Further research is warranted to investigate whether other lifestyle factors (such as diet and physical activity) have a causal role in esophageal malignancy.

## 1. Introduction

Type 2 diabetes (T2D) is a chronic metabolic disorder characterized primarily by hyperglycemia, insulin resistance, and a relative deficiency in insulin secretion.^[1-3]^ According to the World Health Organization (WHO), T2D is the most prevalent form of diabetes globally, posing a significant challenge to public health worldwide.^[4]^ The pathogenesis of T2D is complex, involving an interplay between genetic and environmental factors. Genetic factors^[5-7]^ contribute to the disease by affecting insulin sensitivity and the function of pancreatic β-cell.^[8-10]^ Environmental factors such as unhealthy dietary habits, lack of physical activity, overweight, and obesity significantly increase the risk of developing this disease.^[11,12]^ Additionally, age, ethnicity, and family history are also significant risk factors for T2D.^[13,14]^

Esophageal malignancies are a serious health threat characterized by atypical proliferation of esophageal cells, which may invade surrounding tissues or metastasize distantly.^[15,16]^ Esophageal cancer (EC), including Esophageal squamous cell carcinoma and esophageal adenocarcinoma, represents the most common form of these malignancies.^[17-19]^ There are also some rare pathological types, including gastrointestinal stromal tumors and melanoma, among others.^[20,21]^ The development of these tumors is associated with various risk factors, such as smoking, excessive alcohol consumption, chronic gastroesophageal reflux, and certain dietary habit.^[22,23]^ Treatment options for esophageal malignancies are diverse, including surgery, radiation therapy, chemotherapy, and targeted therapies, with early diagnosis being crucial for improving prognosis.^[24,25]^ Chen et al^[26]^ investigated the relationship between insulin-related traits and prostate cancer, finding that proinsulin may increase the risk of prostate cancer. Mendelian randomization (MR) employs data from genetic studies to estimate the unconfounded relationship between an exposure and an outcome.^[27,28]^ By utilizing genetic variants associated with an exposure, MR can potentially estimate the causal effect of the exposure on the outcome while controlling for confounding factors.^[29]^ Multivariable Mendelian randomization (MVMR) techniques are also used to account for potential pleiotropic effects of genetic instruments, thereby estimating direct effects.^[30,31]^ Yuan et a^[32]^ employed MR analysis to examine the association between diabetes mellitus and prostate cancer risk, demonstrating that diabetes serves as an independent risk factor for prostate cancer. Han et al^[33]^ through a MR phenome-wide association study, revealed that elevated HbA1c levels are associated with increased risks of various conditions including T2D, cataracts, and diabetic nephropathy. The objective of this study is to use MR techniques to reveal the relationship between T2D and the risk of esophageal malignancy. By analyzing the potential impact of genetic variants associated with T2D on esophageal malignancy, our study aims to provide a method that avoids the confounding factors commonly present in traditional observational research, thereby more accurately discerning the causal links between these 2 conditions. This research approach allows us to explore in greater depth how metabolic disorders might influence the risk of malignancies through genetic pathways, providing important scientific evidence for future prevention strategies and therapeutic approaches.

## 2. Materials and methods

### 2.1. Two-sample MR and MVMR analysis

**Figure 1.**
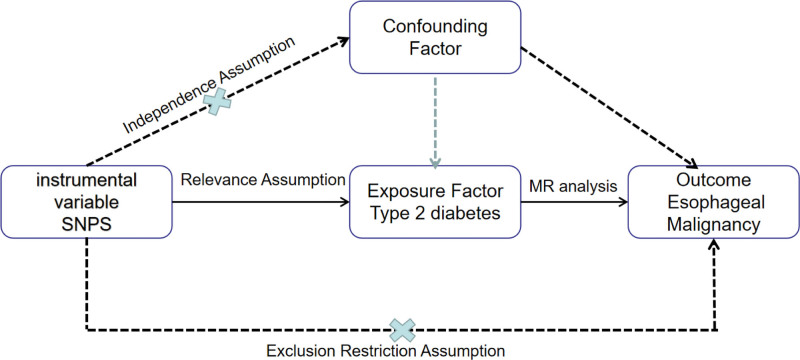
Basic principle of MR research. MR = Mendelian randomization, SNP = single nucleotide polymorphism.

Figure 1 illustrates the fundamental design of our 2-sample Mendelian Randomization (TSMR) study on the potential causal effects of T2D on esophageal malignancy. We compiled summary statistics from publicly accessible databases, selecting single nucleotide polymorphisms (SNPs) strongly associated with T2D and presumed to be randomly assigned at conception, thereby minimizing environmental influences. In the MR analysis, we adhere to 3 core assumptions: the SNPs used as instrumental variables (IVs) must be strongly associated with T2D; IVs must not be related to any potential confounding factors; IVs can only affect the outcome variable,^[34]^ esophageal malignancy, through T2D and not through other pathways. In the preliminary analysis, we utilized data from 2 distinct T2D datasets for MR analysis, employing the standard random-effects inverse variance weighting (IVW) method to estimate the causal impact of T2D on esophageal malignancy. We applied 4 supplementary methods: the weighted median method, the weighted mode method, simple mode, and MR-Egger. This comprehensive approach aids in validating the consistency of the causal estimates. To further validate our hypothesis, we performed a TSMR analysis between HbA1c and esophageal malignancy to examine their linear relationship. To enhance the reliability of our results, we also implemented MVMR to address potential pleiotropy and adjust for confounders such as body mass index (BMI) and hypertension. Our analysis strictly adheres to the STROBE-MR guidelines,^[35]^ ensuring methodological rigor and transparency in our exploration of the potential causal relationship between T2D and esophageal malignancy.

### 2.2. Sources and selection of instrumental variables

We extracted summary data on the associations between T2D and esophageal malignancy from genome-wide association studies. The exposure data set was part of a genome-wide association study (GWAS) catalog focusing on T2D. This dataset was published by Sakaue et al and includes data from a European population.^[36]^ The dataset was used to study the genetic basis of T2D, enabling researchers to identify genetic variants associated with T2D through the analysis of numerous SNPs. Such studies help understand the genetic background of the disease, further advancing the development of preventive and therapeutic methods. The dataset for T2D comorbidities came from a Finnish database. The correction dataset for T2D was maintained jointly by EMBL-EBI^[37]^ and NHGRI (National Human Genome Research Institute).^[38]^ The dataset for esophageal malignancy (ID: malignant neoplasm of esophagus) originated from the Finnish database, containing 232 cases and 1,74,006 controls. Diseases in the Finnish database were diagnosed using ICD codes. The age distribution and inclusion process of patients in the Finnish database can be accessed online through the link https://r11.risteys.finngen.fi/. For detailed information about the data sources, refer to Table 1, which includes information about the datasets and available diagnostic codes.gnancy.

**Table 1:** Information on data included in the study.

Phenotypes/ID	Data source	Study information/PMID	Cases/controls	Author/year
Type 2 diabetes	ebi-a-GCST90018926	European/32005708	38,841/4,51,248	Forgetta/2020
Type 2 diabetes, definitions combined, including avohilmo	finn-b-T2D_INCLAVO	Finnish database	28,717/1,80,722	NA/2022
Type 2 diabetes, wide definition	finn-b-T2D_WIDE	Finnish database	38,617/1,64,552	NA/2022
Type 2 diabetes, strict (exclude DM1)	finn-b-E4_DM2_STRICT	Finnish database	22,876/2,30,293	NA/2022
Type 2 diabetes with other specified/multiple/unspecified complications	finn-b-E4_DM2NASCOMP	Finnish database	10,157/2,43,726	NA/2022
Type 2 diabetes with ophthalmic complications	finn-b-E4_DM2OPTH	Finnish database	7264/2,45,599	NA/2022
Type 2 diabetes with peripheral circulatory complications	finn-b-E4_DM2PERIPH	Finnish database	7144/2,47,809	NA/2022
Type 2 diabetes (SPA correction)	ebi-a-GCST90013942	European/34594039	4,06,831/1,10,39,026	Mbatchou/2021
Type 2 diabetes (firth correction)	ebi-a-GCST90013892	European	4,06,831/1,10,38,957	Mbatchou/2021
Hypertension	ukb-b-12,493	UKB	54,358/4,08,652	Ben Elsworth/2018
Body mass index	ebi-a-GCST90095034	UKB	4,61,460	Ben Elsworth/2018

We employed stringent criteria to select valid and reliable IVs for T2D. Initially, we searched the largest-scale GWAS summary statistics as genetic proxies for T2D. We extracted SNPs strongly associated with T2D as candidate IVs (*P* < 5e−8). Secondly, we excluded SNPs in linkage disequilibrium (*r*^2^ < 0.001) or those with moderate allele frequencies. Thirdly, we excluded SNPs that were unavailable in the outcome GWAS or had proxy SNPs. In this study, we identified BMI and hypertension as confounding factors for esophageal malignancy. To ensure the validity of the selected IVs, we typically used *F*-statistics to assess their strength. The core of the *F*-statistic is to measure the association strength between the IVs and the exposure, thus determining if they are robust enough to provide reliable causal inference. Through the *F*-statistic test, we were able to screen for strong IVs, which not only aids in enhancing the accuracy of causal inference but also strengthens the credibility of the study results. In this paper, we conducted *F*-statistic tests on the selected IVs, *F* = ((N − 2) × (*R*^2^/(1 − *R*^2^)), N represents the sample size and *R*^2^ is the coefficient of determination between the instrumental and exposure variables. ensuring their *F*-values were >10, thus verifying their validity and applicability in the study. Finally, we included 173 qualified SNPs as IVs for the MR analysis. We employed the same method to extract IVs for T2D complications and those adjusted by the Firth correction.

### 2.3. Statistical analysis

In MR studies, we typically use odds ratios (OR) and 95% confidence interval (CI) to present MR estimates. For this study, IVW was chosen as the primary analytical method, supplemented by MR-Egger, weighted median, simple mode, and weighted mode to validate the findings. Using MR-Egger regression and IVW methods. In this study, we selected IVW as the primary analytical method because it utilizes all selected SNPs to calculate a weighted average, minimizing the variance of the estimates and providing efficient and precise causal effect estimates. IVW is suitable when the assumption holds that IVs are not influenced by pleiotropy and there is no significant heterogeneity. Therefore, it is considered the most effective method for causal inference when the assumptions of MR are met. To ensure the robustness of our results, we also employed supplementary methods, including MR-Egger regression, weighted median, and simple mode and weighted mode.^[38]^ The MR-Egger method detects and adjusts for pleiotropy by using a regression model, providing a robust causal effect estimate while examining potential biases and ensuring the reliability of the causal effect estimate. Weighted median estimates the causal effect by taking the weighted median of the effect estimates of multiple IVs, which ensures consistency even if up to 50% of the IVs are biased (e.g., violating MR assumptions). Simple mode and weighted mode are causal effect estimation methods based on the mode, which is robust to outliers and deviations from ideal assumptions. Finally, to better control for confounding factors, particularly known confounders such as BMI and hypertension, we applied MVMR.^[39]^ MVMR allows simultaneous adjustment for multiple exposure variables, providing more accurate causal effect estimates and effectively reducing the influence of confounding factors on the results. It is primarily used to handle situations where IVs may exhibit pleiotropy. Additionally, we utilized Cochrane *Q* for heterogeneity analysis to determine whether there was significant heterogeneity among the causal effect estimates of the selected IVs on the outcome variable. All tests were performed using the “TwoSampleMR” and “devtools” packages in R software (version 4.3.1).

## 3. Results

**Figure 2.**
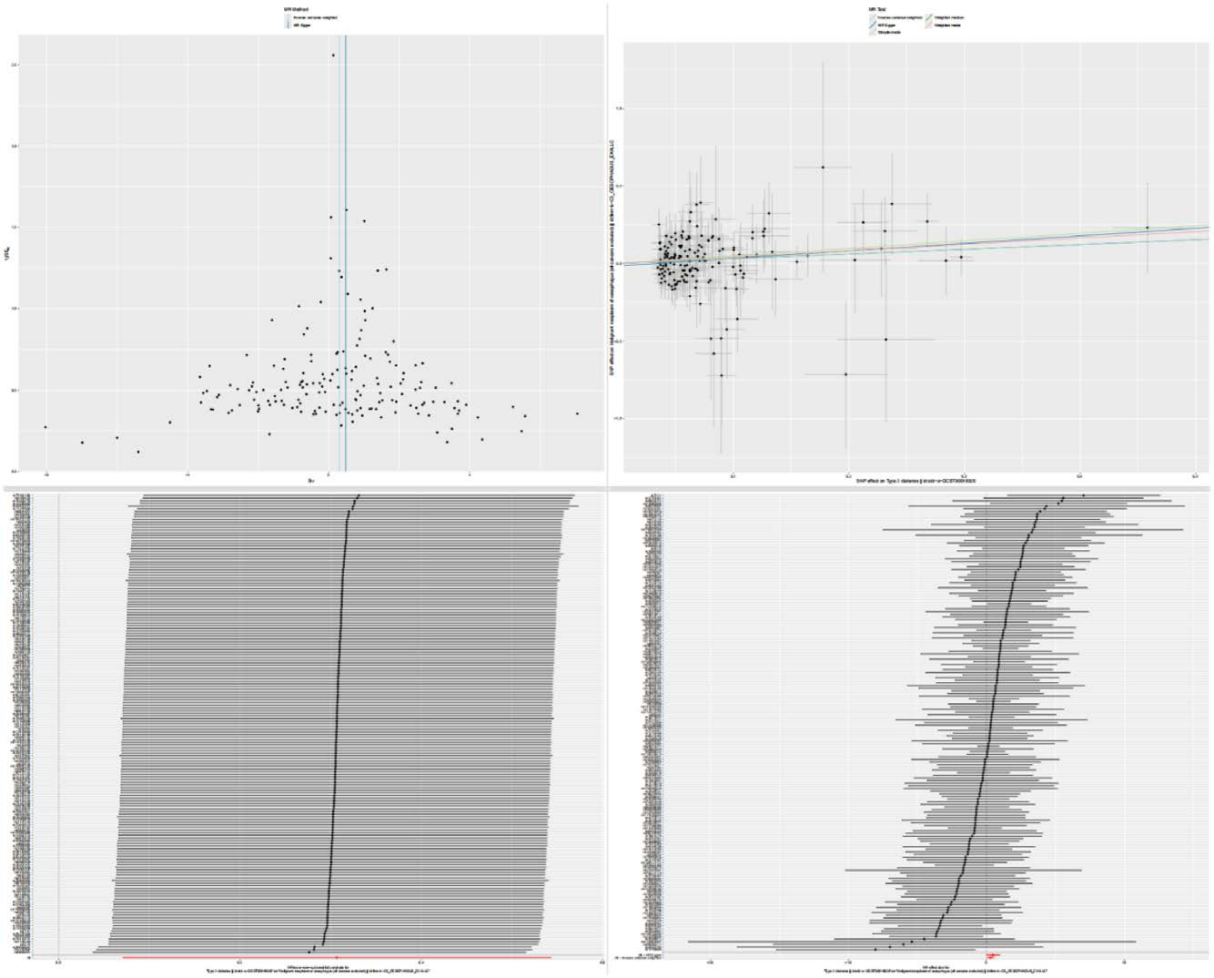
Left top plot: funnel plot; right top plot: scatter plot; left bottom plot: forest plot; right bottom plot: leave-one-out forest plot.

In the MR analysis, given that the IVW method offers the most efficient numerical estimates, the results indicate that T2D significantly increases the risk of esophageal malignancy (IVW, OR = 1.359; 95% CI: 1.073–1.721; *P* = .011). The TSMR analysis supports the causal role of T2D in the development of esophageal malignancy. The Cochran *Q* test and MR-Egger intercept test showed no significant heterogeneity or pleiotropy in the MR analysis results. Furthermore, to assess the impact of each SNP on the overall results, we conducted a leave-one-out sensitivity analysis on the positive results. This method was achieved by sequentially removing each SNP and observing its effect on the overall outcome. The analysis indicated that no single SNP exerted an undue influence on the results, thus ensuring the robustness of the study. To visually display the results of the MR analysis, we created scatter plots. For more information on data analysis and visual presentations, refer to Figure 2, which includes scatter plots for pleiotropy analysis, forest plots using the leave-one-out method, and funnel plots. To further eliminate the impact of confounding factors and pleiotropy, we conducted a MVMR analysis. After adjusting for BMI, hypertension, or both, the statistical significance between T2D and esophageal malignancy persisted (Table 2 for details). For HbA1c analysis, our TSMR revealed no statistically significant association between genetically predicted HbA1c levels and risk of esophageal malignancy. The primary IVW method yielded an OR of 1.43 (95% CI: 0.79–2.61, *P* = .241) per unit increase in HbA1c, suggesting a non-significant positive trend. Sensitivity analyses using MR-Egger (OR = 2.56, *P* = .165), weighted median (OR = 1.87, *P* = .146), and other methods showed broadly consistent but non-significant effect estimates. We found no evidence of heterogeneity (*Q*-test *P* > .57 for all methods) or horizontal pleiotropy (MR-Egger intercept *P* = .326; Table 3).

**Table 2:** Multivariable MR analysis estimating the effect of T2D on esophagus cancer.

Adjustment factor	MVMR		
	Number of SNPs	OR (95% CI)	*P*-value
BMI	151	1.33 (1.04–1.70)	.023
Hypertension	153	1.33 (1.05–1.70)	.017
BMI + hypertension	136	1.31 (1.02–1.69)	.036

BMI = body mass index, CI = confidence interval, MR = Mendelian randomization, MVMR = multivariable Mendelian randomization, OR = odds ratio, SNP = single nucleotide polymorphism.

**Table 3:** TSMR analysis estimating the effect of HbA1c on esophageal malignancy.

Exposure	Outcome	Method	nsnp	*P*val	or	or_lci95	or_uci95
HbA1c	Malignant neoplasm of esophagus	MR-Egger	25	.165	2.557	0.709	9.216
		Weighted median	25	.146	1.874	0.803	4.372
		Inverse variance weighted	25	.241	1.431	0.786	2.607
		Simple mode	25	.695	1.339	0.317	5.654
		Weighted mode	25	.150	2.097	0.790	5.568

MR = Mendelian randomization, SNP = single nucleotide polymorphism, TSMR = 2-sample Mendelian randomization.

**Figure 3.**
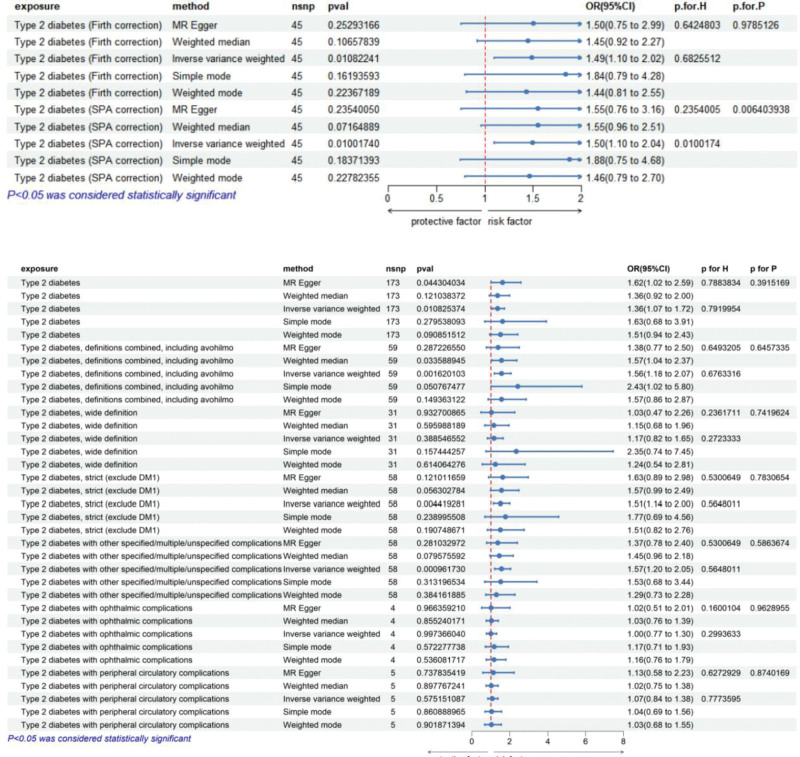
Genetically predicted type 2 diabetes and its complications: associations with the esophageal malignancy IVW, *P* for H (heterogeneity), *P* for (pleiotropy), OR, *P* < .05 was considered statistically significant. CI = confidence interval, IVW = inverse variance weighted, MR = Mendelian randomization, OR = odds ratio.

To understand the relationship between different subgroups of T2D and esophageal malignancy, we analyzed data from the Finnish database, which provides the most comprehensive records of T2D complications. Data from both SPA- and Firth-corrected T2D showed a causal association with esophageal malignancy. In subgroup analyses of T2D with complications, specifically those with ophthalmic complications and peripheral circulatory complications, the number of qualifying SNPs was insufficient to reveal a causal relationship with esophageal malignancy. With wide range of T2D complications T2D was statistically significant (*P* < .01, OR = 1.57) Among all these subgroup analyses, no significant heterogeneity or pleiotropy was observed, except in the analysis using the wide definition of the T2D database, which includes individuals treated with hypoglycemic medications even without a clear T2D diagnosis but with symptoms of hyperglycemia. The robustness of the results was confirmed using leave-one-out analysis and MR-PRESSO tests (Fig. 3).

## 4. Discussion

Based on a TSMR analysis using large-scale GWAS data, we studied the causal relationship between T2D and esophageal malignancy. We found that genetic susceptibility to T2D is associated with an increased risk of esophageal malignancy. The relationship between T2D and esophageal malignancy suggests that early intervention for diabetes could reduce the incidence and severity of esophageal malignancy, potentially alleviating long-term health burdens and improving patient prognosis.

A meta-analysis by Wang et al^[40]^ included 11 prospective cohort studies and 6 case-control studies, all of which reported an association between diabetes and the risk of EC, In analysis of all these 17 studies that reported relative risk of DM and EC, the SRRs and corresponding 95% CIs were 1.30 (95% CI: 1.12–1.50) in a random-effects model for those with diabetes compared with non-diabetic individuals. There was border significant heterogeneity among studies (*Q* = 26.93, *P* = .042, *I*^2^ = 40.6%). Further analysis revealed that the risk of EC increased in diabetic men, whereas this association was not observed in diabetic women.

Dixon et al^[41]^ investigated the association between T2D and esophageal adenocarcinoma (EAC). Through a retrospective analysis of the US Veterans Affairs database from 2005 to 2009, researchers found that diabetic patients had a significantly higher risk of developing esophageal adenocarcinoma compared to patients with only gastroesophageal reflux disease (GERD). Specifically, the diagnosis of diabetes was significantly associated with the risk of EC, with adjusted results showing that diabetic patients had 2.2 times the risk of EC compared to GERD patients. Additionally, an Australian study^[42]^ compared the prevalence of T2D in patients with EAC and control groups, indicating that T2D increases the risk of EAC. Particularly, when patients had both T2D and obesity, the OR was as high as 3.5, while the OR for T2D alone was 1.86 and for obesity alone was 2.67.

However, the exact mechanisms by which esophageal malignancies develop in diabetic patients remain unclear, although several hypotheses have been proposed. Hyperinsulinemia caused by T2D can promote tumor initiation and progression through various mechanisms, including enhancing cell proliferation, inhibiting apoptosis, and increasing cancer cell survival.

The specific mechanisms involve signal transduction mediated by the insulin receptor, insulin-like growth factor 1 receptor, and their heterodimers. These signaling pathways can activate the PI3K/Akt and Ras/MAPK pathways, further promoting cell growth and division.^[43]^ Additionally, T2DM is often accompanied by chronic inflammation, which alters the microenvironment to favor tumor initiation and progression. Dysregulation of adipose tissue leads to abnormal secretion of leptin and adiponectin, hormones that also play roles in cancer development.^[44]^

T2D is considered a potential risk factor for GERD.^[45,46]^ Studies have found that GERD symptoms are more common in T2D patients than in non-diabetics, possibly due to diabetes-induced delayed gastric emptying and lower esophageal sphincter dysfunction. Additionally, T2D patients exhibit higher rates of esophageal inflammation and motility disorders, increasing GERD risk, which is a significant risk factor for EAC. GERD can promote the development of esophageal adenocarcinoma through various mechanisms. Some studies suggest that metformin use can reduce the risk of certain cancers in T2D patients.^[47]^

This anticancer effect may be due to metformin’s ability to activate AMP-activated protein kinase in PC-3 cell lines, inhibiting protein synthesis in tumor cells and reducing insulin levels.^[48]^ However, the use of antidiabetic drugs has not yet been proven to reduce the incidence of EC. Given that HbA1c serves as a well-established biomarker for long-term glycemic control in diabetes management, we specifically examined its association with esophageal malignancy risk based on previous epidemiological evidence. Several large-scale observational studies^[49-52]^ have consistently reported that elevated HbA1c levels, even within the prediabetic range, are associated with increased risks of various cancers. This biological plausibility stems from multiple mechanisms: chronic hyperglycemia may promote tumorigenesis through formation of advanced glycation end-products that induce oxidative stress, glucose-mediated activation of inflammatory pathways, and metabolic reprogramming in cancer cells (the Warburg effect). Our MR approach extends these observational findings by assessing whether this relationship reflects a causal association. While our primary analysis established a causal link between T2D and esophageal malignancy, we further investigated the role of glycemic control through TSMR analysis of genetically predicted HbA1c levels. The results revealed no statistically significant association between HbA1c and esophageal malignancy risk (IVW OR = 1.43, 95% CI: 0.79–2.61, *P* = .241), with consistent null findings across sensitivity analyses (MR-Egger, weighted median). This suggests 3 potential explanations: the observed T2D–EC association may be mediated through pathways independent of chronic hyperglycemia, such as insulin signaling or chronic inflammation; the glycemic control threshold required for cancer risk modulation may exceed the physiological range captured by common HbA1c-associated SNPs; or the limited statistical power due to the modest case number (n = 232) in the FinnGen EC GWAS may have constrained our ability to detect a true effect.

This study has several significant advantages. Firstly, this is the first study to use large-scale GWAS data to investigate the causal relationship between T2D and esophageal malignancy. The TSMR method can effectively overcome several limitations of traditional observational studies, such as reverse causality, confounding factors, and various biases, providing more reliable causal inference. Secondly, all IVs used in this MR analysis were rigorously selected, with the lowest *F*-value still exceeding 10, ensuring the precision of the analysis results. Additionally, we employed various methods to test sensitivity, horizontal pleiotropy, and heterogeneity. The results of these tests indicate that the association between T2D and esophageal malignancy is stable and robust. However, this study has some limitations. Firstly, all study subjects in the GWAS were of European descent, which may limit the generalizability of our findings. Future research covering more diverse populations and regions will help verify whether this causal relationship is universal. Although we used IVW and MR-PRESSO global tests to detect and adjust for confounding factors like hypertension and BMI, there may still be uncontrolled confounders such as participants’ education level, personality, and nutritional status, which could affect the accuracy of the results.

Additionally, although the MR analysis of T2D subgroups with complications allows for a comprehensive understanding of the associations between various complications and esophageal malignancy, it is important to acknowledge that there is considerable sample overlap between the exposure and outcome datasets, which could increase the risk of type I errors.

Finally, a key limitation of our study is the reliance on open-source databases, which lack detailed patient-level clinical phenotypic data, including pathology reports and disease staging. As a result, we were unable to perform precise subgroup analyses based on critical clinical measures such as HbA1c levels or conduct nonlinear MR analysis. These limitations may impact the depth and accuracy of our findings, especially in exploring the complex interactions between metabolic disorders and esophageal malignancy. To address these gaps, our next steps involve collaborating with clinical institutions to collect more comprehensive patient data, including clinical and pathological information. We also plan to explore additional validation methods to strengthen the robustness of our findings, which could lead to a more nuanced understanding of the causal relationships explored in this study.

## Acknowledgments

We would like to thank the participants and researchers of the FinnGen study, the UK Biobank, and the IEU Open GWAS Project. We sincerely thank all the researchers who contributed valuable genetic linkage statistical data to this study.

## Author contributions

**Conceptualization:** Mingzhi Lin, Yiming Hui, Haitian Li.

**Data curation:** Mingzhi Lin, Chongrui Li, Bin Li, Zhongting Li.

**Formal analysis:** Mingzhi Lin, Chongrui Li, Bin Li.

**Methodology:** Mingzhi Lin.

**Project administration:** Mingzhi Lin.

**Validation:** Yuzhen Chen.

**Visualization:** Yiming Hui, Haitian Li, Zhizhong Zheng.

**Writing – original draft:** Mingzhi Lin.

**Writing – review & editing:** Mingzhi Lin, Bin Li.
